# Analysis of Long Non-Coding RNA-Mediated Regulatory Networks of *Plutella xylostella* in Response to *Metarhizium anisopliae* Infection

**DOI:** 10.3390/insects13100916

**Published:** 2022-10-09

**Authors:** Junaid Zafar, Junlin Huang, Xiaoxia Xu, Fengliang Jin

**Affiliations:** Key Laboratory of Bio-Pesticide Innovation and Application of Guangdong Province, College of Plant Protection, South China Agricultural University, Guangzhou 510642, China

**Keywords:** lncRNA, insect immunity, host–pathogen, lepidoptera, bio-control, entomopathogenic fungus, fat body

## Abstract

**Simple Summary:**

*Plutella xylostella* is one of the most destructive insect pests of cruciferous crops worldwide. Notorious for its ability to resist a myriad of chemical insecticides, this pest has become a nuisance, leading scientists to probe for alternative eco-friendly control measures, such as *Metarhizium anisopliae*, an insect pathogenic fungus. In response to fungal infection, insects mount a wide array of immune responses mediated by several regulatory molecules, including long non-coding RNAs (lncRNAs). Evidence suggests that lncRNAs are significantly induced in response to pathogenic infection in plants and animals. However, their role during insect host–pathogen interactions is still in its infancy. In the current study, we employed a strand-specific RNA sequencing technique to decipher the role of lncRNAs in the *P. xylostella* fat body during *M. anisopliae* infection. Our findings will provide a genetic resource for future functional studies of lncRNAs and shed light on understanding insect–pathogen interactions. These findings would be helpful in designing pest management strategies via gene silencing technologies.

**Abstract:**

Long non-coding RNAs (lncRNAs) represent a diverse class of RNAs that are structurally similar to messenger RNAs (mRNAs) but do not encode proteins. Growing evidence suggests that in response to biotic and abiotic stresses, the lncRNAs play crucial regulatory roles in plants and animals. However, the potential role of lncRNAs during fungal infection has yet to be characterized in *Plutella xylostella*, a devastating pest of cruciferous crops. In the current study, we performed a strand-specific RNA sequencing of *Metarhizium anisopliae*-infected (Px36hT, Px72hT) and uninfected (Px36hCK, Px72hCK) *P. xylostella* fat body tissues. Comprehensive bioinformatic analysis revealed a total of 5665 and 4941 lncRNAs at 36 and 72-h post-infection (hpi), including 563 (Px36hT), 532 (Px72hT) known and 5102 (Px36hT), 4409 (Px72hT) novel lncRNA transcripts. These lncRNAs shared structural similarities with their counterparts in other species, including shorter exon and intron length, fewer exon numbers, and a lower expression profile than mRNAs. LncRNAs regulate the expression of neighboring protein-coding genes by acting in a *cis* and *trans* manner. Functional annotation and pathway analysis of *cis*-acting lncRNAs revealed their role in several immune-related genes, including *Toll*, *serpin*, *transferrin*, *βGRP* etc. Furthermore, we identified multiple lncRNAs acting as microRNA (miRNA) precursors. These miRNAs can potentially regulate the expression of mRNAs involved in immunity and development, suggesting a crucial lncRNA–miRNA-mRNA complex. Our findings will provide a genetic resource for future functional studies of lncRNAs involved in *P. xylostella* immune responses to *M. anisopliae* infection and shed light on understanding insect host–pathogen interactions.

## 1. Introduction

Non-coding RNAs (ncRNAs) are the transcripts that do not encode proteins. They constitute the largest class of RNAs and are arbitrarily divided into small ncRNAs (sncRNAs) and long ncRNAs (lncRNAs) [1,2]. Even though 90% of the eukaryotic genome is transcribed, approximately 2% can encode proteins, demonstrating that a significant proportion embodies ncRNAs [3]. The lncRNAs are transcripts with a length of ≥200 nucleotides (nt) [4]. LncRNAs, like messenger RNAs (mRNAs), are transcribed by RNA polymerase II or III, spliced and capped at 5′ ends [5]. Contrary to mRNAs, lncRNAs are expressed at low levels and in highly spatiotemporal patterns, generally showing poor conservation across species [6]. Based on their genomic location, lncRNAs can be classified into intergenic, intronic, sense, and antisense [7]. The lncRNAs play essential roles in several biological processes, such as epigenetics [8], dosage compensation [9], alternative splicing [10], cell cycle [11], and gene expression regulation [12]. Several lncRNAs have been functionally characterized in plants and animals, though the functions of lncRNAs, particularly in insects, remain unclear.

In insects, *Drosophila melanogaster* became the first species to have lncRNAs identified from RNA-seq data [13], allowing subsequent studies to reveal their role in the development of the nervous system [14] and spermatogenesis [15,16]. Some of the functionally characterized lncRNAs among insects include *yellow-achaete intergenic RNA* (sleeping behavior) [17], *roX1* and *roX2* (dosage compensation) in *Drosophila* [18], *AncR-1* (neuronal functions) in *Apis mellifera* [19], and *iab-1* (physiological processes) in *Bombyx mori* [20]. Under stress, the lncRNAs have been reported to play a significant role [21]. In silkworms, several lncRNAs were identified to be involved in the immune responses to *B. mori* nucleopolyhedrovirus (BmNPV) infection [22]. During viral infection in *A. mellifera* and *Apis cerana*, 11 lincRNAs (long intergenic RNAs) were differentially expressed, indicating their potential role in the defence mechanism [23]. The *lncRNA-CR46018* modulates immunity by positively regulating the Toll pathway in *Drosophila*, through interaction with Dif/Dorsal, during bacterial infection [24]. Since the functional evidence of lncRNAs among insects is still in its infancy, transcriptomic investigations provide a valuable understanding and basis for future research.

The diamondback moth (DBM), *Plutella xylostella* (Lepidoptera: Plutellidae), is one of the most damaging pests of cruciferous crops worldwide, with an annual management cost of more than 4 billion US dollars [25]. One of the primary reasons for its continuous success against contemporary pest management approaches is its ability to develop resistance to various chemical toxins and growth regulators [26,27]. *Metarhizium anisopliae,* an entomopathogenic fungus, provides an ecologically safe alternative to conventional chemicals for the control of susceptible, resistant and multi-resistant insect pests [28]. The *M. anisopliae* infection begins when an insect comes in contact with anthropogenically dispersed conidia found in the soil [29]. Once the fungus has gained entry, the fungal hyphae accumulate in the fat body. The fat body is a multifunctional and dynamic tissue [30] involved in several biosynthetic activities, including immune responses [31]. In the fat body, immune genes are induced by microbial invasion and they encode antimicrobial peptides (AMPs), which are then released into the hemolymph for further action [32], suggesting the crucial role of the fat body in insect immunity. These immune responses are governed by a variety of ncRNAs, including lncRNAs. For instance, the *lncRNA-CR33942* is abundantly expressed in the fat body tissues of *Drosophila* and positively regulates Imd immune responses [33,34]. Understanding the regulatory roles of lncRNAs in insects may lead to the development of new pest management tactics.

To systematically identify the lncRNAs and their regulatory networks, we performed a whole transcriptome strand-specific RNA sequencing of *M. anisopliae*-infected and uninfected fat body tissues from 3rd instar *P. xylostella* larvae at 36 (sub-lethal) and 72 (lethal) hpi (hours post infection). Results showed that fungus stress resulted in differential expression of multiple lncRNAs over both time points, suggesting the host response to infection. Additional analysis revealed that lncRNAs could interact with other regulatory molecules (mRNA & sncRNA) involved in host immune responses and play crucial regulatory roles, presenting multilayer regulatory networks.

These results will serve as the basis for deciphering the underlying molecular mechanisms involved in insect-pathogen interactions while also providing a tool for the creation of effective eco-friendly pest management strategies.

## 2. Materials and Methods

### 2.1. Fungal Infection and RNA Sequencing

Susceptible 3rd instar larvae of *P. xylostella* were topically infected with the entomopathogenic fungus *M. anisopliae*, whereas aqueous 0.05% Tween-80 (Sigma-P1754) was taken as a control [35]. Fat body tissues from uninfected (Px36hCK and Px72hCK) and *M. anisopliae*-infected (Px36hT and Px72hT) larvae were dissected in phosphate buffer saline (PBS) using a sterilized dissection kit and snap-frozen in liquid nitrogen. Total RNA was extracted using the TRIzol reagent kit (Invitrogen, Carlsbad, CA, USA) following the manufacturer’s instructions. The concentration and integrity of RNA were determined using NanoDrop™ (Thermo Fisher, Wilmington, DE, USA) and the Agilent 2100 Bioanalyzer (Agilent, Santa Clara, CA, USA), respectively. After total RNA was extracted, ribosomal RNAs (rRNAs) were removed to retain mRNAs and ncRNAs. Subsequently, libraries were constructed and sequenced using the Illumina HiSeq^TM^ 4000 by Gene Denovo Biotechnology Co. (Guangzhou, China).

### 2.2. Read Filters, Assembly and lncRNA Identification

To obtain high-quality clean data, raw reads were filtered using fastp (version 18.0) [36] to remove reads containing adaptors, more than 10% of unknown nucleotides (N), more than 50% low-quality (Q-value ≤ 20) bases and contaminants. The clean reads were then mapped to the rRNA database to remove the remaining rRNA reads using a short-read alignment tool Bowtie2 (version 2.2.8) [37]. High-quality clean reads were then mapped to the *P. xylostella* genome (GCA_000330985.1) using HISAT2 (version 2.1.0) [38], and transcripts were assembled using StringTie (version 1.3.4) [39] following a reference-based approach [40]. Transcripts with lengths ≥ 200bp were retained for further analysis. We used Cuffcompare to annotate and compare novel transcripts by aligning them to reference genome. Transcripts with one of the class codes “u, i, x, c, e, o” were defined as novel transcripts. To assess the protein-coding potential, two software packages, Coding-Non-Coding Index (CNCI) (version 2) and Coding Potential Calculator (CPC2) [41], were used [42]. Transcripts revealing coding potential with a score of CNCI > 0 and CPC2 > 0 were all removed. The intersection of CNCI and CPC2 was considered reliable for lncRNA results. The transcripts expression level was normalized to fragment per kilobase million (FPKM), which eliminates the influence of transcripts lengths and sequencing data amount on the calculation of transcripts expression.

### 2.3. LncRNA Classification

Predicted lncRNA sequences were annotated against the Rfam database (http://rfam.org; accessed 10 December 2021) using Infernal (v1.1.2) [43] to classify them into various ncRNA families, in which each family is represented by a multiple sequence alignment (MSA), a consensus secondary structure (CSS), and a covariance model (CM) [44]. Putative lncRNAs were classified into five classes according to their location relative to protein-coding genes: sense lncRNAs, antisense lncRNAs, intronic lncRNAs, bidirectional lncRNAs and intergenic lncRNAs. Different kinds of lncRNAs may have various biological functions.

### 2.4. Screening of Differentially Expressed lncRNAs

Differential expression analysis between two groups was performed using DESeq2 [45]. The transcripts with a parameter of false discovery rate (FDR) < 0.05, fold change value ≥ 1 and an adjusted *p* value (q-value) ≤ 0.05 were considered as differentially expressed lncRNAs.

### 2.5. Prediction of Pre-miRNAs and Target Genes of lncRNAs

To find potential microRNA (miRNA) precursors (pre-miRNAs), lncRNAs were aligned to miRbase (www.mirbase.org; accessed 10 December 2021) and hits with coverage of more than 90% were selected. Additionally, a support vector machine (SVM) based software, miRPara (version 6.3), was used to predict miRNA precursors [46].

LncRNAs interact with target genes mainly in the *cis* and *trans* manner [47]. The *cis*-acting lncRNAs are involved in the regulation of their adjacent protein-coding genes. In the current study, we searched for the genes located within 10 kb upstream or downstream of the lncRNAs for functional investigations. The *trans*-lncRNA regulation is based on interaction with co-expressed genes. RNAplex was used to calculate the binding energy between lncRNA-mRNA duplexes [48]. Subsequently, the Gene Ontology (GO) database and Kyoto Encyclopaedia of Genes & Genomes (KEGG) pathway enrichment analyses of all the predicted genes were performed for functional annotation. The corrected Q value ≤ 0.05 was set as the threshold to determine significant enrichment of the gene sets.

## 3. Results

To obtain a comprehensive understanding of the dynamic responses of lncRNAs in *P. xylostella* infected with *M. anisopliae*, the fat body tissues were dissected at 36 hpi and 72 hpi and subjected to RNA-seq analysis.

### 3.1. Sequencing and Identification of lncRNAs

A total of 1,504,866,764 clean reads were obtained from 12 libraries. The clean reads were mapped to the reference genome (GCA_000330985.1), and the results showed that mapping ratios ranged from 61.55% to 69.37%, whereas uniquely mapped reads accounted for 57.27% to 63.85% (Table 1). A rigorous filtering process was performed to remove low-quality lncRNA transcripts, which resulted in the identification of several known and novel lncRNAs (Figure S1). We identified 563 (Px36hT) and 532 (Px72hT) known lncRNAs.

**Figure 1:**
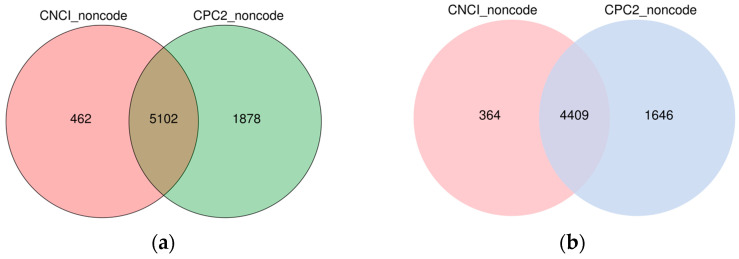
Venn Diagram of novel lncRNAs by coding capacity analysis at different time courses. (**a**) 36 h post-infection. (**b**) 72 h post-infection. CNCI indicates Coding-Non-Coding-Index and CPC2 indicates Coding Potential Calculator.

The coding capacity analysis of these transcripts predicted 5102 novel lncRNAs at 36 hpi and 4409 novel lncRNA transcripts at 72 hpi (Figure 1).

### 3.2. Genomic Characterization of the lncRNAs

**Figure 2:**
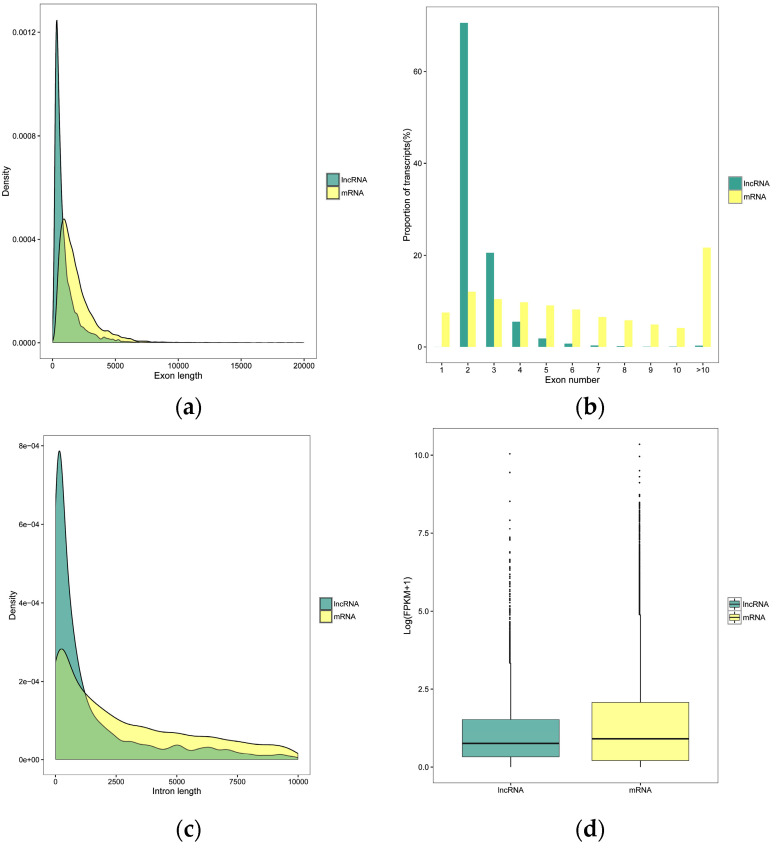

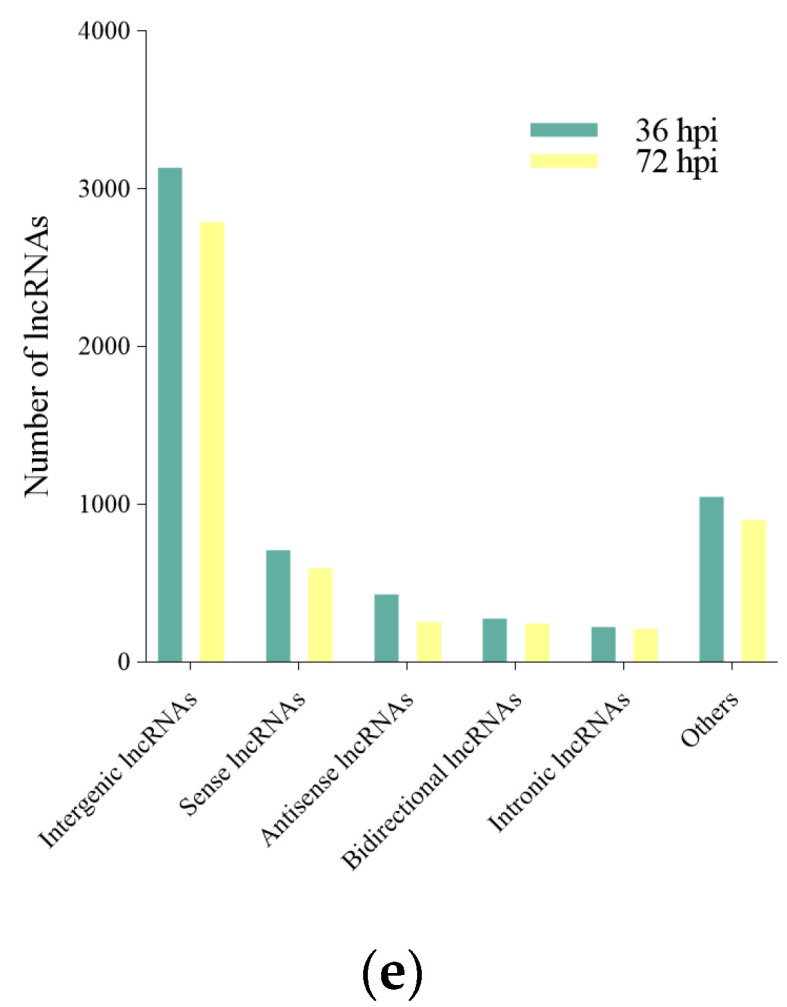
Characteristics of *P. xylostella* lncRNAs. (**a**) Exon lengths of lncRNAs and mRNAs. (**b**) Number of exons in each lncRNA and mRNA. (**c**) Size distributions of introns for lncRNAs and mRNAs. (**d**) Expression levels of lncRNAs and mRNAs. (**e**) LncRNA family analysis.

To understand the genomic characteristics of the lncRNAs expressed in the *M. anisopliae* infected fat body tissues, we performed a comparative analysis of the exon number, intron and exon length of all mRNAs and lncRNAs identified in the current study. Compared to mRNAs, these lncRNAs were shorter in exon and intron length and had fewer exon numbers (Figure 2A–C), as described in previous studies [49,50,51]. Furthermore, the expression analysis of transcripts showed that the expression levels of lncRNAs were lower than mRNAs (Figure 2D). Based on their relation to neighbouring protein-coding genes: 3132, 707, 426, 275, and 221 (Px36hCK vs. Px36hT) and 2787, 595, 256, 245, and 211 (Px72hCK vs. Px72hT) were classified as intergenic, sense, antisense, bidirectional, and intronic lncRNAs, respectively (Figure 2E).

### 3.3. Identification of Differentially Expressed lncRNAs

The objective of the current study was to identify the lncRNAs that respond to *M. anisopliae* infection at various time points. The differential expression analysis revealed eight lncRNAs in Px36hCK vs. Px36hT, including six up-regulated and two down-regulated lncRNAs (Table S1); whereas ten differentially expressed lncRNAs were identified in Px72hCK vs. Px72hT, including six up-regulated and four down-regulated lncRNAs (Table S2). *XR_960741.1*, *MSTRG.9405.1* and *MSTRG.10357.3* (intergenic lncRNAs) were highly expressed, while *MSTRG.22113.1*, a *cis*-regulatory intergenic type of lncRNA expression, was significantly suppressed at 36 hpi. Similarly, at 72 hpi, the lncRNAs *MSTRG.20086.1* (intergenic), *MSTRG.33965.4* (sense) and *XR_960679.1* (intergenic) were significantly expressed, whereas the expression of *MSTRG.22373.2* (sense) and *MSTRG.33331.1* (intergenic) was significantly reduced, followed by *M. anisopliae* infection. It is worth mentioning that amongst differentially expressed lncRNAs, an intergenic lncRNA-*XR_960679.1* was constantly up-regulated at both time points. These results show an increase in differentially expressed transcripts as the *M. anisopliae* infection progressed, indicating a critical role of lncRNAs in *P. xylostella* response to fungal infection.

### 3.4. Functional Analysis of M. anisopliae-Responsive lncRNA Targets in P. xylostella

In the current study, we identified 1275 mRNAs as potential targets of 1051 differentially expressed *cis*-regulatory lncRNAs, resulting in 1469 pairings (649 upstream & 820 downstream) in Px36hCK vs. Px36hT (Table S3). GO analysis showed the putative target genes of these *cis*-regulatory lncRNAs annotated in 19 biological functions (i.e., metabolic processes, cellular processes, single organism processes, signalling, behaviour and developmental processes), seven molecular functions (i.e., catalytic activity, binding, and transporter activity), and 13 cellular components (i.e., cell part, cell junction, and membrane). The top 20 GO terms are presented in Table S4. Similarly, in Px72hCK vs. Px72hT, a total of 945 pairings (426 upstream & 519 downstream) were identified between 676 differentially expressed *cis*-regulatory lncRNAs and 829 mRNAs (Table S5). GO analysis showed 15 biological processes (i.e., localization, response to stimulus and biological regulation), eight molecular functions (antioxidant activity and signal transducer) and 14 cellular components (cell, membrane part and macromolecular complex). The top 20 GO terms are presented in Table S6. KEGG pathway analysis in five categories (metabolism, cellular processes, environmental information processing, organismal system and genetic information processing) revealed that 149 pathways are enriched within lncRNA targets and related to metabolic pathways (122), biosynthesis of secondary metabolites (39), oxidative phosphorylation (18), microbial metabolism (15), lysosome (09), Hippo signaling pathway (05), and immune system (01) in Px36hCK vs. Px36hT. The top 20 significantly enriched pathways are presented in Table S7. Similarly, in Px72hCK vs. Px72hT, a total of 103 pathways enriched within lncRNA targets were found, including biosynthesis of antibiotics (14), lysosome (09), RNA transport (07), metabolism of xenobiotics by cytochrome P450 (02), and MAPK signaling pathway (01). The top 20 significantly enriched pathways are presented in Table S8. Furthermore, we filtered *cis*-regulatory lncRNAs with their immune-related target genes to identify their potential involvement in immune responses during *M. anisopliae* infection. We found that differentially expressed *cis*-lncRNAs-mRNA pairings are involved in crucial immune responses and pathways. *MSTRG.8010.1* is involved in upstream regulation of beta-1,3-glucan-binding protein (pathogen recognition), *MSTRG.35418.1* in downstream regulation of Toll-like receptor 3 (modulator of the immune response), *MSTRG.47817.1* in upstream regulation of serpin (melanization and antimicrobial peptide), and *MSTRG.29984.1* is involved in targeting cytochrome P450 (detoxification of xenobiotics). The list of differentially expressed immunity-related *cis*-acting lncRNAs and their target genes is provided in Table 2.

Additionally, lncRNAs can regulate the expression of mRNAs by acting in *trans* [3]. Therefore, we studied the lncRNAs by screening mRNAs as potential *trans*-regulatory targets of all lncRNAs. GO analysis revealed the putative targets of these lncRNAs in Px36hCK vs. Px36hT, which were annotated as 22 biological processes terms (i.e., immune system, response to stimulus and biological regulation), 18 cellular component-related terms (i.e., cell part, extracellular region), and 10 molecular functions (i.e., catalytic activity, signal transducer). The top 20 GO terms are presented in Table S9. Similar GO analysis performed in Px72hCK vs. Px72hT annotated the target genes mainly in biological processes related terms (i.e., immune system, behaviour, metabolic process), molecular function (i.e., binding, transporter activity) and cellular components (i.e., cell part and macromolecular complex). The top 20 GO terms are presented in Table S10. KEGG pathway analysis in five categories (metabolism, cellular processes, environmental information processing, organismal system and genetic information processing) revealed 212 pathways enriched within lncRNA targets and are related to metabolic pathways (696), biosynthesis of secondary metabolites (236), oxidative phosphorylation (120), microbial metabolism (101), lysosome (89) and RNA transport (56) in Px36hCK vs. Px36hT. The top 20 significantly enriched pathways are presented in Table S11. Similarly, in Px72hCK vs. Px72hT, a total of 203 pathways enriched within lncRNA targets were found, including biosynthesis of secondary metabolites (207), lysosome (84), RNA transport (70), and metabolism of xenobiotics by cytochrome P450 (37). The top 20 significantly enriched pathways are presented in Table S12.

### 3.5. Pre-miRNA Analysis of lncRNAs in P. xylostella

**Figure 3:**
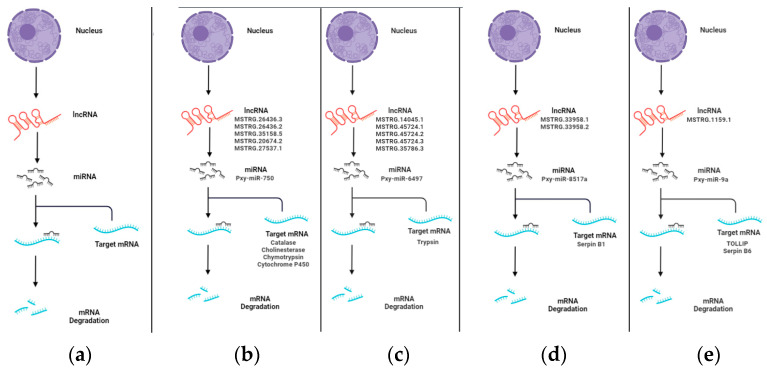
LncRNA-miRNA-mRNA complex. The lncRNA acts as a miRNA precursor, which may participate in mRNA degradation. Key immunity and developmental-related complex are presented here. (**a**) The proposed model of lncRNA acting as miRNA precursor. (**b**) Multiple lncRNAs as the precursor of pxy-miR-750, which can regulate the expression of multiple mRNAs such as *catalase*, *cholinesterase*, *chymotrypsin* and *cytochrome p450*. (**c**) Multiple lncRNAs as the precursor of pxy-miR-6497 whose target site was identified in *trypsin*. (**d**) LncRNAs *MSTRG.33958.1/2* as precursors of pxy-8517a which can target *serpin B1*; and (**e**) LncRNA *MSTRG.1159.1* as a potential precursor of pxy-miR-9a whose target sites were identified in *tollip* and *serpin B6*.

Recent genomic investigations have suggested that a significant fraction of lncRNAs may serve as precursors of miRNAs [52,53]. To investigate whether the lncRNAs in *M. anisopliae*-infected fat body tissues of *P. xylostella* are precursors of miRNAs, the lncRNA sequences were subjected to BLAST analysis to miRBase. In total, 17 lncRNAs were identified as precursors of nine miRNAs in Px36hCK vs. Px36hT, including four lncRNAs as potential precursors of pxy-mir-6497 and three lncRNAs as precursors of pxy-mir-750 and two lncRNAs, each as precursors of pxy-mir-8497 and pxy-mir-8517a, respectively. Similarly, nine lncRNAs were identified from Px72hCK vs. Px72hT serving as potential precursors of seven miRNAs, including pxy-mir-8497, pxy-mir-750, pxy-mir-8517a and pxy-mir-6497. Interestingly, three miRNAs (pxy-mir-8497, pxy-mir-6497, and pxy-mir-750) were shared between both groups. The potential lncRNAs identified in our study as miRNA precursors are presented in Tables S13 and S14. We filtered these miRNAs through our previous studies to identify their potential immunity and development-related target genes [54,55] and identified several important genes, including *trypsin*, *serpin B1*, *catalase*, *cholinesterase*, *chymotrypsin,* and *cytochrome p450*. A schematic diagram of the lncRNA-miRNA-mRNA complex is presented in Figure 3, particularly focusing on immune-related genes.

## 4. Discussion

In the last decade, lncRNAs have garnered global attention for their critical regulatory roles and several studies have been performed on plants and mammals [3,56]. However, the information regarding insect lncRNAs is relatively limited. Advancements in high-throughput techniques have enabled researchers to identify lncRNAs in various insect species, such as *D. melanogaster* [13], *B. mori* [57], *A. mellifera* [23], *Anopheles gambiae* [58], *Aedes aegypti* [59], and *Nilopervata lugens* [60]. In *P. xylostella*, a few lncRNA studies have been performed [61], but most focus on exploring the role of lncRNAs in insecticide resistance [51,62,63,64]. Several studies have been conducted in *P. xylostella* investigating the role of miRNAs-mRNAs in response to pathogenic fungus infection [35,54,65]. However, little is known about the characterization and functions of lncRNAs involved in the interaction between *P. xylostella* and pathogenic fungi.

In the current study, we used rRNA removal and strand-specific RNA sequencing to methodically profile and identify lncRNAs involved in the responses of *P. xylostella* to *M. anisopliae* infection. We identified 5665 (563 known and 5102 novel) and 4941 (532 known and 4409 novel) lncRNAs from control and *M. anisopliae*-infected fat body tissues of *P. xylostella* at 36 and 72 h, respectively. Genomic characteristics revealed that these lncRNAs shared features similar to other species, including shorter exon and intron length, fewer exon numbers and lower expression levels [60,66,67,68], suggesting that these lncRNA features are common in different species. It can be stated that these sets of lncRNAs will be beneficial for further functional studies. However, since the lncRNAs are often expressed in tissue or stage-specific patterns [69], the identified transcripts in our study could only be a fraction, and many more lncRNAs can be discovered using different tissues and pathogens.

LncRNAs manipulate gene expression under biotic and abiotic stresses [21,53]. For instance, in sacbrood virus (SBV) infected honeybees, 15 lincRNAs showed differential expression [23]. Similarly, in BmNPV-infected silkworm larvae, several differentially expressed lncRNAs were observed and validated using qRT-PCR [22]. In our study, we identified several differentially expressed lncRNAs in the *M. anisopliae*-infected fat body of *P. xylostella*. These results are supported by similar findings where *Nosema ceranae*, a microsporidium, infected the midguts of *A. cerana* and significantly changed the expression levels of lncRNAs at 7 and 10 days post-infection (dpi), suggesting that the fungal infection significantly altered lncRNA expression levels [50].

Unlike mRNAs, lncRNAs have little functional information available. Studies have suggested that lncRNAs can target protein-coding genes via *cis* or *trans* regulation [70]. Our results identified multiple differentially expressed *cis*-acting lncRNAs and their target mRNAs. Functional annotations suggested that the target genes of differentially expressed *cis*-regulatory lncRNAs in Px36hCK vs. Px36hT and Px72hCK vs. Px72hT were involved in 39 and 37 functional terms, respectively. The objective of the current research was to identify the lncRNAs that target immune-related genes. Recent studies have shown that besides mRNAs, an abundance of miRNAs and lncRNAs affect immune responses during host–pathogen interactions [71,72]. Our analyses identified multiple differentially expressed *cis*-acting lncRNAs that target crucial immune genes, such as *βGRP*, *Toll-like receptors 3*/*6*, *trypsin*, *transferrin*, *serpins,* and *cytochrome P450* during *M. anisopliae* infection. These genes are crucial to *P. xylostella* immune responses during fungal infection, as described in previous studies [73]. In *B. mori*, a differentially expressed *cis*-acting lncRNA, *lncRNA4,* was identified from BmNPV-infected midgut and fat body tissues. Expression analysis showed that the *lncRNA4* followed a similar expression pattern as Toll and might act as a decoy and titrate away dimerization of Toll on the membrane, thus preventing its activation [74]. Similarly, in *D. melanogaster,* lncRNA *CR46018* and *lincRNA-IBIN* were significantly induced upon *Micrococcus luteus* infection and were involved in the Toll pathway regulation [75]. These findings support our studies in which we identified lncRNA *MSTRG.35418.1* regulating *toll-like receptor 3* (Gene ID: 105390974) via *cis*-regulation, implying the crucial role of lncRNAs in host immunity during pathogen infection.

LncRNAs can act as potential precursors of miRNA; the sheared miRNA can target its respective mRNA and result in degradation [76,77,78]. In this study, we identified 17 (36 h) and nine (72 h) lncRNAs as potential precursors of several miRNAs. Among these miRNAs, miR-9a is involved in *P. xylostella* immune response to fungal infection [73], miR-6497 is highly expressed in BmNPV infected silkworms [22], and miR-274 inhibition facilitates *B. mori* cytoplasmic polyhedrosis virus (BmCPV) replication. Some miRNAs had multiple precursor lncRNAs, e.g., pxy-miR-750 expressed at both time intervals (36 h & 72 h) has five precursors lncRNAs. The pxy-miR-750 is known to be involved in many biological processes, including the development, resistance and immunity [79]. These results presented a multi-layered (lncRNA-miRNA-mRNA) immune response during pathogen infection.

## 5. Conclusions

In conclusion, our study identified 5665 (563 known and 5102 novel) and 4941 (532 known and 4409 novel) lncRNAs in the *M. anisopliae*-infected fat body tissues of *P. xylostella* at 36 and 72 h, including multiple differentially expressed transcripts. The results show that fungal infection could significantly alter the expression of host lncRNAs. These lncRNAs were likely to participate in immune responses to pathogen infection by modulating gene expression in the *cis* and *trans* manner or acting as miRNA precursors. Our results could provide the foundation for further functional studies of lncRNAs crucial to host–pathogen interactions.

## Figures and Tables

**Table 1:** Statistics of read align to reference genome.

Sample	Total Clean Reads	Total Mapped Ratio	Uniquely Mapped Ratio
Px36hCK1	114,737,868	62.05%	57.27%
Px36hCK2	112,126,214	62.72%	57.90%
Px36hCK3	110,573,374	62.87%	57.96%
Px36hT1	111,791,770	62.26%	61.41%
Px36hT2	110,615,464	64.95%	60.18%
Px36hT3	110,957,412	62.37%	60.48%
Px72hCK1	141,895,296	69.37%	63.85%
Px72hCK2	156,208,658	66.09%	62.39%
Px72hCK3	123,592,924	66.95%	61.72%
Px72hT1	139,702,358	64.70%	60.16%
Px72hT2	135,959,518	61.55%	62.61%
Px72hT3	136,705,908	66.94%	62.90%

**Table 2:** Immune-related *cis*-acting lncRNAs and their target genes.

lncRNA	mRNA	Up/Down	Target Description
MSTRG.35418.1	ncbi_105390974	DOWNSTREAM	toll receptor 3
MSTRG.31934.1	ncbi_105389601	UPSTREAM	toll receptor 6
MSTRG.42048.1	ncbi_105393670	DOWNSTREAM	Transferrin
MSTRG.47817.1	ncbi_105396589	UPSTREAM	serpin B6
MSTRG.40967.1	ncbi_105393232	UPSTREAM	Chymotrypsin
MSTRG.38482.1	ncbi_105392199	UPSTREAM	Trypsin
MSTRG.33197.1	ncbi_105390096	UPSTREAM	JNK-interacting protein 1
MSTRG.33658.1	ncbi_105390273	DOWNSTREAM	ryanodine receptor 44F-like
MSTRG.21279.1	ncbi_105385460	UPSTREAM	NF-kappa-B-activating protein
MSTRG.29984.1	ncbi_105388787	UPSTREAM	cytochrome P450
MSTRG.8010.1	MSTRG.8013	UPSTREAM	beta-1,3-glucan-binding protein
MSTRG.2041.1	MSTRG.2044	UPSTREAM	trypsin-like serine proteinase 2

## Data Availability

The data supporting this study’s findings are available from the corresponding author upon reasonable request.

## Supplementary Materials

The following supporting information can be downloaded at: https://www.mdpi.com/article/10.3390/insects13100916/s1, Figure S1: The lncRNA analysis pipeline; Table S1: Differentially expressed lncRNAs in *Metarhizium anisopliae* infected fat body tissues of *Plutella xylostella* at Px36hCK vs. Px36hT; Table S2: Differentially expressed lncRNAs in *Metarhizium anisopliae* infected fat body tissues of *Plutella xylostella* at Px72hCK vs. Px72hT; Table S3: Differentially expressed *cis*-lncRNAs in *Metarhizium anisopliae* infected fat body tissues of *Plutella xylostella* at Px36hCK vs. Px36hT; Table S4: Top 20 GO categories enriched by *cis*-regulatory target genes of lncRNAs in Px36hCK vs. Px36hT; Table S5: Differentially expressed *cis*-lncRNAs in *Metarhizium anisopliae* infected fat body tissues of *Plutella xylostella* at Px72hCK vs. Px72hT; Table S6: Top 20 GO categories enriched by *cis*-regulatory target genes of lncRNAs in Px72hCK vs. Px72hT; Table S7: Top 20 pathways enriched by *cis*-regulatory target genes of lncRNAs in Px36hCK vs. Px36hT; Table S8: Top 20 pathways enriched by *cis*-regulatory target genes of lncRNAs in Px72hCK vs. Px72hT; Table S9: Top 20 GO categories enriched by *trans*-regulatory target genes of lncRNAs in Px36hCK vs. Px36hT; Table S10: Top 20 GO categories enriched by *trans*-regulatory target genes of lncRNAs in Px72hCK vs. Px72hT; Table S11: Top 20 pathways enriched by *trans*-regulatory target genes of lncRNAs in Px36hCK vs. Px36hT; Table S12: Top 20 pathways enriched by *trans*-regulatory target genes of lncRNAs in Px72hCK vs. Px72hT; Table S13: Prediction of lncRNAs as the precursor of miRNAs in *Metarhizium anisopliae*-infected *Plutella xylostella* fat body tissues at Px36hCK vs. Px36hT; Table S14: Prediction of lncRNAs as the precursor of miRNAs in *Metarhizium anisopliae*-infected *Plutella xylostella* fat body tissues at Px72hCK vs. Px72hT.

## References

[1] Mattick J.S., Makunin I.V. (2006). Non-coding RNA. Hum. Mol. Genet..

[2] Quinn J.J., Chang H.Y. (2016). Unique features of long non-coding RNA biogenesis and function. Nat. Rev. Genet..

[3] Jarroux J., Morillon A., Pinskaya M., Rao M.R.S. (2017). History, Discovery, and Classification of lncRNAs. Long Non Coding RNA Biology.

[4] Wu H., Yang L., Chen L.-L. (2017). The diversity of long noncoding RNAs and their generation. Trends Genet..

[5] Tamtaji O.R., Derakhshan M., Rashidi Noshabad F.Z., Razaviyan J., Hadavi R., Jafarpour H., Jafari A., Rajabi A., Hamblin M.R., Mahabady M.K. (2022). Non-Coding RNAs and Brain Tumors: Insights Into Their Roles in Apoptosis. Front. Cell Dev. Biol..

[6] Kaushik K., Leonard V.E., Kv S., Lalwani M.K., Jalali S., Patowary A., Joshi A., Scaria V., Sivasubbu S. (2013). Dynamic expression of long non-coding RNAs (lncRNAs) in adult zebrafish. PLoS ONE.

[7] Guttman M., Rinn J.L. (2012). Modular regulatory principles of large non-coding RNAs. Nature.

[8] Wang C., Wang L., Ding Y., Lu X., Zhang G., Yang J., Zheng H., Wang H., Jiang Y., Xu L. (2017). LncRNA structural characteristics in epigenetic regulation. Int. J. Mol. Sci..

[9] Sahakyan A., Yang Y., Plath K. (2018). The role of Xist in X-chromosome dosage compensation. Trends Cell Biol..

[10] Gonzalez I., Munita R., Agirre E., Dittmer T.A., Gysling K., Misteli T., Luco R.F. (2015). A lncRNA regulates alternative splicing via establishment of a splicing-specific chromatin signature. Nat. Struct. Mol. Biol..

[11] Zhang L., Kang W., Lu X., Ma S., Dong L., Zou B. (2018). LncRNA CASC11 promoted gastric cancer cell proliferation, migration and invasion in vitro by regulating cell cycle pathway. Cell Cycle.

[12] Engreitz J.M., Haines J.E., Perez E.M., Munson G., Chen J., Kane M., McDonel P.E., Guttman M., Lander E.S. (2016). Local regulation of gene expression by lncRNA promoters, transcription and splicing. Nature.

[13] Young R.S., Marques A.C., Tibbit C., Haerty W., Bassett A.R., Liu J.-L., Ponting C.P. (2012). Identification and properties of 1119 candidate lincRNA loci in the *Drosophila melanogaster* genome. Genome Biol. Evol..

[14] Li K., Tian Y., Yuan Y., Fan X., Yang M., He Z., Yang D. (2019). Insights into the Functions of LncRNAs in *Drosophila*. Int. J. Mol. Sci..

[15] Maeda R.K., Sitnik J.L., Frei Y., Prince E., Gligorov D., Wolfner M.F., Karch F. (2018). The lncRNA male-specific abdominal plays a critical role in *Drosophila* accessory gland development and male fertility. PLoS Genet..

[16] Vedelek V., Bodai L., Grézal G., Kovács B., Boros I.M., Laurinyecz B., Sinka R. (2018). Analysis of *Drosophila melanogaster* testis transcriptome. BMC Genom..

[17] Soshnev A.A., Ishimoto H., McAllister B.F., Li X., Wehling M.D., Kitamoto T., Geyer P.K. (2011). A conserved long noncoding RNA affects sleep behavior in *Drosophila*. Genetics.

[18] Kim M., Faucillion M.-L., Larsson J. (2018). *RNA-on-X 1* and *2* in *Drosophila melanogaster* fulfill separate functions in dosage compensation. PLoS Genet..

[19] Sawata M., Takeuchi H., Kubo T. (2004). Identification and analysis of the minimal promoter activity of a novel noncoding nuclear RNA gene, *AncR-1*, from the honeybee (*Apis mellifera* L.). RNA.

[20] Wang H., Hu H., Xiang Z., Lu C., Dai F., Tong X. (2019). Identification and characterization of a new long noncoding RNA *iab-1* in the Hox cluster of silkworm, *Bombyx mori* identification of *iab-1*. J. Cell. Biochem..

[21] Valadkhan S., Valencia-Hipólito A., Morris K.V. (2016). lncRNAs in Stress Response. Long Non-Coding RNAs in Human Disease.

[22] Zhang S., Yin H., Shen M., Huang H., Hou Q., Zhang Z., Zhao W., Guo X., Wu P. (2020). Analysis of lncRNA-mediated gene regulatory network of *Bombyx mori* in response to BmNPV infection. J. Invertebr. Pathol..

[23] Jayakodi M., Jung J.W., Park D., Ahn Y.-J., Lee S.-C., Shin S.-Y., Shin C., Yang T.-J., Kwon H.W. (2015). Genome-wide characterization of long intergenic non-coding RNAs (lincRNAs) provides new insight into viral diseases in honey bees *Apis cerana* and *Apis mellifera*. BMC Genom..

[24] Zhou H., Ni J., Wu S., Ma F., Jin P., Li S. (2021). *lncRNA-CR46018* positively regulates the *Drosophila* Toll immune response by interacting with Dif/Dorsal. Dev. Comp. Immunol..

[25] Zalucki M.P., Shabbir A., Silva R., Adamson D., Shu-Sheng L., Furlong M.J. (2012). Estimating the economic cost of one of the world’s major insect pests, *Plutella xylostella* (Lepidoptera: Plutellidae): Just how long is a piece of string?. J. Econ. Entomol..

[26] Talekar N., Shelton A. (1993). Biology, ecology, and management of the diamondback moth. Annu. Rev. Entomol..

[27] Sun J., Liang P., Gao X. (2012). Cross-resistance patterns and fitness in fufenozide-resistant diamondback moth, *Plutella xylostella* (Lepidoptera: Plutellidae). Pest Manag. Sci..

[28] Zimmermann G. (2007). Review on safety of the entomopathogenic fungus *Metarhizium anisopliae*. Biocontrol. Sci. Technol..

[29] Hesketh H., Roy H., Eilenberg J., Pell J., Hails R. (2010). Challenges in modelling complexity of fungal entomopathogens in semi-natural populations of insects. BioControl.

[30] Skowronek P., Wójcik Ł., Strachecka A. (2021). Fat body—Multifunctional insect tissue. Insects.

[31] Buchon N., Silverman N., Cherry S. (2014). Immunity in *Drosophila melanogaster*—From microbial recognition to whole-organism physiology. Nat. Rev. Immunol..

[32] Hoffmann J.A. (1995). Innate immunity of insects. Curr. Opin. Immunol..

[33] Zhou H., Wu S., Liu L., Li R., Jin P., Li S. (2022). *Drosophila* Relish Activating *lncRNA-CR33942* Transcription Facilitates Antimicrobial Peptide Expression in Imd Innate Immune Response. Front. Immunol..

[34] Krause S.A., Overend G., Dow J.A., Leader D.P. (2022). FlyAtlas 2 in 2022: Enhancements to the *Drosophila melanogaster* expression atlas. Nucleic Acids Res..

[35] Zafar J., Shoukat R.F., Zhang Y., Freed S., Xu X., Jin F. (2020). *Metarhizium anisopliae* Challenges Immunity and Demography of *Plutella xylostella*. Insects.

[36] Chen S., Zhou Y., Chen Y., Gu J. (2018). fastp: An ultra-fast all-in-one FASTQ preprocessor. Bioinformatics.

[37] Langmead B., Salzberg S.L. (2012). Fast gapped-read alignment with Bowtie 2. Nat. Methods.

[38] Kim D., Langmead B., Salzberg S.L. (2015). HISAT: A fast spliced aligner with low memory requirements. Nat. Methods.

[39] Pertea M., Pertea G.M., Antonescu C.M., Chang T.-C., Mendell J.T., Salzberg S.L. (2015). StringTie enables improved reconstruction of a transcriptome from RNA-seq reads. Nat. Biotechnol..

[40] Martin J.A., Wang Z. (2011). Next-generation transcriptome assembly. Nat. Rev. Genet..

[41] Kang Y.-J., Yang D.-C., Kong L., Hou M., Meng Y.-Q., Wei L., Gao G. (2017). CPC2: A fast and accurate coding potential calculator based on sequence intrinsic features. Nucleic Acids Res..

[42] Han S., Liang Y., Li Y., Du W. (2016). Long noncoding RNA identification: Comparing machine learning based tools for long noncoding transcripts discrimination. Biomed Res. Int..

[43] Nawrocki E.P., Eddy S.R. (2013). Infernal 1.1: 100-fold faster RNA homology searches. Bioinformatics.

[44] Joshi R.K., Megha S., Basu U., Rahman M.H., Kav N.N. (2016). Genome wide identification and functional prediction of long non-coding RNAs responsive to *Sclerotinia sclerotiorum* infection in *Brassica napus*. PLoS ONE.

[45] Love M.I., Huber W., Anders S. (2014). Moderated estimation of fold change and dispersion for RNA-seq data with DESeq2. Genome Biol..

[46] Wu Y., Wei B., Liu H., Li T., Rayner S. (2011). MiRPara: A SVM-based software tool for prediction of most probable microRNA coding regions in genome scale sequences. BMC Bioinform..

[47] Fatica A., Bozzoni I. (2014). Long non-coding RNAs: New players in cell differentiation and development. Nat. Rev. Genet..

[48] Tafer H., Hofacker I.L. (2008). RNAplex: A fast tool for RNA–RNA interaction search. Bioinformatics.

[49] Zhou Q.-Z., Zhang B., Yu Q.-Y., Zhang Z. (2016). BmncRNAdb: A comprehensive database of non-coding RNAs in the silkworm, *Bombyx mori*. BMC Bioinform..

[50] Chen D., Chen H., Du Y., Zhou D., Geng S., Wang H., Wan J., Xiong C., Zheng Y., Guo R. (2019). Genome-wide identification of long non-coding RNAs and their regulatory networks involved in *Apis mellifera ligustica* response to *Nosema ceranae* infection. Insects.

[51] Liu F., Guo D., Yuan Z., Chen C., Xiao H. (2017). Genome-wide identification of long non-coding RNA genes and their association with insecticide resistance and metamorphosis in diamondback moth, *Plutella xylostella*. Sci. Rep..

[52] Fejes-Toth K., Sotirova V., Sachidanandam R., Assaf G., Hannon G.J., Kapranov P., Foissac S., Willingham A.T., Duttagupta R., Dumais E. (2009). Post-transcriptional processing generates a diversity of 5′-modified long and short RNAs. Nature.

[53] Meng X., Li A., Yu B., Li S. (2021). Interplay between miRNAs and lncRNAs: Mode of action and biological roles in plant development and stress adaptation. Comput. Struct Biotechnol. J..

[54] Shakeel M., Xu X., Xu J., Li S., Yu J., Zhou X., Xu X., Hu Q., Yu X., Jin F. (2018). Genome-wide identification of Destruxin A-responsive immunity-related microRNAs in diamondback moth, *Plutella xylostella*. Front. Immunol..

[55] Zafar J., Zhang Y., Huang J., Freed S., Shoukat R.F., Xu X., Jin F. (2021). Spatio-Temporal Profiling of *Metarhizium anisopliae*—Responsive microRNAs Involved in Modulation of *Plutella xylostella* Immunity and Development. J. Fungi.

[56] Liu J., Wang H., Chua N.H. (2015). Long noncoding RNA transcriptome of plants. Plant Biotechnol. J..

[57] Wu Y., Cheng T., Liu C., Liu D., Zhang Q., Long R., Zhao P., Xia Q. (2016). Systematic identification and characterization of long non-coding RNAs in the silkworm, *Bombyx mori*. PLoS ONE.

[58] Jenkins A.M., Waterhouse R.M., Kopin A.S., Muskavitch M.A. (2014). Long non-coding RNA discovery in *Anopheles gambiae* using deep RNA sequencing. BioRxiv.

[59] Etebari K., Asad S., Zhang G., Asgari S. (2016). Identification of *Aedes aegypti* long intergenic non-coding RNAs and their association with *Wolbachia* and dengue virus infection. PLOS Negl. Trop. Dis..

[60] Xiao H., Yuan Z., Guo D., Hou B., Yin C., Zhang W., Li F. (2015). Genome-wide identification of long noncoding RNA genes and their potential association with fecundity and virulence in rice brown planthopper, *Nilaparvata lugens*. BMC Genom..

[61] Wang Y., Xu T., He W., Shen X., Zhao Q., Bai J., You M. (2018). Genome-wide identification and characterization of putative lncRNAs in the diamondback moth, *Plutella xylostella* (L.). Genomics.

[62] Zhu B., Li L., Wei R., Liang P., Gao X. (2021). Regulation of GSTu1-mediated insecticide resistance in *Plutella xylostella* by miRNA and lncRNA. PLoS Genet..

[63] Zhu B., Xu M., Shi H., Gao X., Liang P. (2017). Genome-wide identification of lncRNAs associated with chlorantraniliprole resistance in diamondback moth *Plutella xylostella* (L.). BMC Genom..

[64] Etebari K., Furlong M.J., Asgari S. (2015). Genome wide discovery of long intergenic non-coding RNAs in Diamondback moth (*Plutella xylostella*) and their expression in insecticide resistant strains. Sci. Rep..

[65] Xu J., Xu X., Li S., Wang S., Xu X., Zhou X., Yu J., Yu X., Shakeel M., Jin F. (2017). Genome-wide profiling of *Plutella xylostella* immunity-related miRNAs after *Isaria fumosorosea* infection. Front. Physiol..

[66] Gupta P., Peter S., Jung M., Lewin A., Hemmrich-Stanisak G., Franke A., von Kleist M., Schütte C., Einspanier R., Sharbati S. (2019). Analysis of long non-coding RNA and mRNA expression in bovine macrophages brings up novel aspects of *Mycobacterium avium* subspecies paratuberculosis infections. Sci. Rep..

[67] Jenkins A.M., Waterhouse R.M., Muskavitch M.A. (2015). Long non-coding RNA discovery across the genus *anopheles* reveals conserved secondary structures within and beyond the Gambiae complex. BMC Genom..

[68] Brown J.B., Boley N., Eisman R., May G.E., Stoiber M.H., Duff M.O., Booth B.W., Wen J., Park S., Suzuki A.M. (2014). Diversity and dynamics of the *Drosophila* transcriptome. Nature.

[69] Li M., Liu L. (2015). Neural functions of long noncoding RNAs in *Drosophila*. J. Comp. Physiol..

[70] Knauss J.L., Sun T. (2013). Regulatory mechanisms of long noncoding RNAs in vertebrate central nervous system development and function. Neuroscience.

[71] Agliano F., Rathinam V.A., Medvedev A.E., Vanaja S.K., Vella A.T. (2019). Long Noncoding RNAs in Host-Pathogen Interactions. Trends Immunol..

[72] Moure U.A.E., Tan T., Sha L., Lu X., Shao Z., Yang G., Wang Y., Cui H. (2022). Advances in the Immune Regulatory Role of Non-Coding RNAs (miRNAs and lncRNAs) in Insect-Pathogen Interactions. Front. Immunol..

[73] Xu J., Xu X., Shakeel M., Li S., Wang S., Zhou X., Yu J., Xu X., Yu X., Jin F. (2017). The entomopathogenic fungi *Isaria fumosorosea* plays a vital role in suppressing the immune system of *Plutella xylostella*: RNA-Seq and DGE analysis of immunity-related genes. Front. Microbiol..

[74] Satyavathi V., Ghosh R., Subramanian S. (2017). Long Non-Coding RNAs Regulating Immunity in Insects. Noncoding RNA.

[75] Valanne S., Salminen T.S., Järvelä-Stölting M., Vesala L., Rämet M. (2019). Immune-inducible non-coding RNA molecule *lincRNA-IBIN* connects immunity and metabolism in *Drosophila melanogaster*. PLoS Pathog..

[76] Venkatesh J., Wasson M.-C.D., Brown J.M., Fernando W., Marcato P. (2021). LncRNA-miRNA axes in breast cancer: Novel points of interaction for strategic attack. Cancer Lett..

[77] Chen L., Zhou Y., Li H. (2018). LncRNA, miRNA and lncRNA-miRNA interaction in viral infection. Virus Res..

[78] Guo L., Zhao Y., Yang S., Zhang H., Wu Q., Chen F. (2014). An integrated evolutionary analysis of miRNA–lncRNA in mammals. Mol. Biol. Rep..

[79] Zhu B., Li X., Liu Y., Gao X., Liang P. (2017). Global identification of microRNAs associated with chlorantraniliprole resistance in diamondback moth *Plutella xylostella* (L.). Sci. Rep..

