# Supplementary material for: Analysis of Long Non-Coding RNA-Mediated Regulatory Networks of Plutella xylostella in Response to Metarhizium anisopliae Infection
Source: Insects. 2022 Oct 9;13(10):916. doi: 10.3390/insects13100916 (PMC9604237; doi:10.3390/insects13100916)
Supplement: Supplementary file 1 [file insects-13-00916-s001.zip › Table S10 Top 20 GO categories enriched by trans-regulatory target genes of lncRNAs in Px72hCK vs. Px72hT.pdf]

Table S10 Top 20 GO categories enriched by *trans*-regulatory target genes of lncRNAs in Px72hCK vs Px72hT.

GO term	Number of enriched genes
Catalytic activity	434
Metabolic process	407
Cellular process	398
Single-organism process	356
Binding	346
Cell	224
Cell part	224
Organelle	155
Membrane	152
Biological regulation	131
Localization	127
Macromolecular complex	119
Regulation of biological process	117
Membrane part	111
Response to stimulus	87
Cellular component organization or biogenesis	69
Organelle part	67
Transporter activity	47
Signaling	46
Developmental process	39
