# Supplementary material for: Analysis of Long Non-Coding RNA-Mediated Regulatory Networks of Plutella xylostella in Response to Metarhizium anisopliae Infection
Source: Insects. 2022 Oct 9;13(10):916. doi: 10.3390/insects13100916 (PMC9604237; doi:10.3390/insects13100916)
Supplement: Supplementary file 1 [file insects-13-00916-s001.zip › Table S11 Top 20 pathways enriched by trans-regulatory target genes of lncRNAs in Px36hCK vs Px36hT.pdf]

Table S11 Top 20 pathways enriched by *trans*-regulatory target genes of lncRNAs in Px36hCK vs Px36hT

Pathway	Number of enriched genes
Metabolic pathways	696
Biosynthesis of secondary metabolites	236
Biosynthesis of antibiotics	133
Oxidative phosphorylation	120
Microbial metabolism in diverse environments	101
Lysosome	89
Purine metabolism	89
Neuroactive ligand-receptor interaction	75
Carbon metabolism	75
Peroxisome	69
Phagosome	65
Protein processing in the endoplasmic reticulum	62
Ubiquitin mediated proteolysis	61
Spliceosome	57
RNA transport	56
Biosynthesis of amino acids	55
Fatty acid metabolism	52
Phosphatidylinositol signaling system	47
Endocytosis	47
FoxO signaling pathway	45
