# Supplementary material for: Analysis of Long Non-Coding RNA-Mediated Regulatory Networks of Plutella xylostella in Response to Metarhizium anisopliae Infection
Source: Insects. 2022 Oct 9;13(10):916. doi: 10.3390/insects13100916 (PMC9604237; doi:10.3390/insects13100916)
Supplement: Supplementary file 1 [file insects-13-00916-s001.zip › Table S12 Top 20 pathways enriched by trans-regulatory target genes of lncRNAs in Px72hCK vs Px72hT.pdf]

Table S12 Top 20 pathways enriched by *trans*-regulatory target genes of lncRNAs in Px72hCK vs Px72hT

Pathway	Number of enriched genes
Metabolic pathways	600
Biosynthesis of secondary metabolites	207
Biosynthesis of antibiotics	118
Oxidative phosphorylation	110
Purine metabolism	100
Lysosome	84
Microbial metabolism in diverse environments	76
RNA transport	70
Neuroactive ligand-receptor interaction	69
Protein processing in the endoplasmic reticulum	66
Spliceosome	65
Carbon metabolism	63
Peroxisome	58
Phagosome	56
Ribosome biogenesis in eukaryotes	47
Biosynthesis of amino acids	47
Glutathione metabolism	44
Fatty acid metabolism	44
Endocytosis	41
Metabolism of xenobiotics by cytochrome P450	37
