# Supplementary material for: Analysis of Long Non-Coding RNA-Mediated Regulatory Networks of Plutella xylostella in Response to Metarhizium anisopliae Infection
Source: Insects. 2022 Oct 9;13(10):916. doi: 10.3390/insects13100916 (PMC9604237; doi:10.3390/insects13100916)
Supplement: Supplementary file 1 [file insects-13-00916-s001.zip › Table S4 Top 20 GO categories enriched by cis-regulatory target genes of lncRNAs in Px36hCK vs. Px36hT.pdf]

Table S4 Top 20 GO categories enriched by *cis*-regulatory target genes of lncRNAs in Px36hCK vs. Px36hT.

GO term	Number of enriched genes
Metabolic process	84
Catalytic activity	79
Cellular process	72
Single-organism process	66
Binding	54
Cell	45
Cell part	45
Membrane	30
Organelle	28
Membrane part	24
Localization	22
Biological regulation	22
Response to stimulus	21
Regulation of biological process	20
Macromolecular complex	18
Signaling	14
Transporter activity	11
Organelle part	10
Biogenesis	9

Signal transducer activity	7
