# Supplementary material for: Analysis of Long Non-Coding RNA-Mediated Regulatory Networks of Plutella xylostella in Response to Metarhizium anisopliae Infection
Source: Insects. 2022 Oct 9;13(10):916. doi: 10.3390/insects13100916 (PMC9604237; doi:10.3390/insects13100916)
Supplement: Supplementary file 1 [file insects-13-00916-s001.zip › Table S6 Top 20 GO categories enriched by cis-regulatory target genes of lncRNAs in Px72hCK vs. Px72hT.pdf]

Table S6 Top 20 GO categories enriched by *cis*-regulatory target genes of lncRNAs in Px72hCK vs. Px72hT.

GO term	Number of enriched genes
Metabolic process	49
Catalytic activity	49
Cellular process	44
Single-organism process	41
Binding	34
Membrane	24
Cell	21
Cell part	21
Localization	18
Membrane part	17
Organelle	14
Biological regulation	12
Response to stimulus	11
Regulation of biological process	11
Macromolecular complex	6
Transporter activity	6
Signaling	5
Organelle part	5
Biogenesis	4

Antioxidant activity	3
