# Supplementary material for: Analysis of Long Non-Coding RNA-Mediated Regulatory Networks of Plutella xylostella in Response to Metarhizium anisopliae Infection
Source: Insects. 2022 Oct 9;13(10):916. doi: 10.3390/insects13100916 (PMC9604237; doi:10.3390/insects13100916)
Supplement: Supplementary file 1 [file insects-13-00916-s001.zip › Table S7 Top 20 pathways enriched by cis-regulatory target genes of lncRNAs in Px36hCK vs. Px36hT.pdf]

Table S7 Top 20 pathways enriched by *cis*-regulatory target genes of lncRNAs in Px36hCK vs. Px36hT.

Pathway	Number of enriched genes
Metabolic pathways	122
Biosynthesis of secondary metabolites	39
Oxidative phosphorylation	18
Endocytosis	17
Biosynthesis of antibiotics	16
Microbial metabolism in diverse environments	15
Neuroactive ligand-receptor interaction	14
Fatty acid metabolism	13
Purine metabolism	13
Peroxisome	12
ABC transporters	11
Fatty acid biosynthesis	10
Spliceosome	10
Protein processing in endoplasmic reticulum	10
Carbon metabolism	10
Phagosome	9
Ubiquitin mediated proteolysis	9
Lysosome	9
Porphyrin and chlorophyll metabolism	8

ECM-receptor interaction	8
