# Supplementary material for: Analysis of Long Non-Coding RNA-Mediated Regulatory Networks of Plutella xylostella in Response to Metarhizium anisopliae Infection
Source: Insects. 2022 Oct 9;13(10):916. doi: 10.3390/insects13100916 (PMC9604237; doi:10.3390/insects13100916)
Supplement: Supplementary file 1 [file insects-13-00916-s001.zip › Table S8 Top 20 pathways enriched by cis-regulatory target genes of lncRNAs in Px72hCK vs. Px72hT.pdf]

Table S8 Top 20 pathways enriched by *cis*-regulatory target genes of lncRNAs in Px72hCK vs. Px72hT.

Pathway	Number of enriched genes
Metabolic pathways	79
Biosynthesis of secondary metabolites	32
Oxidative phosphorylation	14
Biosynthesis of antibiotics	14
Neuroactive ligand-receptor interaction	12
Carbon metabolism	11
Microbial metabolism in diverse environments	11
Lysosome	9
Purine metabolism	9
Protein processing in endoplasmic reticulum	8
Endocytosis	8
Aminoacyl-tRNA biosynthesis	7
Glycerolipid metabolism	7
Glutathione metabolism	7
Fatty acid metabolism	7
RNA transport	7
Sphingolipid metabolism	6
Glycerophospholipid metabolism	6
Wnt signaling pathway	6

Biosynthesis of amino acids	6
