# Supplementary material for: Analysis of Long Non-Coding RNA-Mediated Regulatory Networks of Plutella xylostella in Response to Metarhizium anisopliae Infection
Source: Insects. 2022 Oct 9;13(10):916. doi: 10.3390/insects13100916 (PMC9604237; doi:10.3390/insects13100916)
Supplement: Supplementary file 1 [file insects-13-00916-s001.zip › Table S9 Top 20 GO categories enriched by trans-regulatory target genes of lncRNAs in Px36hCK vs. Px36hT.pdf]

Table S9 Top 20 GO categories enriched by *trans*-regulatory target genes of lncRNAs in Px36hCK vs Px36hT.

GO term	Number of enriched genes
Catalytic activity	487
Metabolic process	455
Cellular process	425
Single-organism process	371
Binding	347
Cell	218
Cell part	218
Membrane	168
Organelle	137
Biological regulation	136
Regulation of the biological process	127
Localization	123
Membrane part	120
Macromolecular complex	113
Response to stimulus	84
Signaling	65
Organelle part	59
Cellular component organization or biogenesis	53
Transporter activity	53
Multicellular organismal process	32
