# Supplementary figures and images for: Analysis of Long Non-Coding RNA-Mediated Regulatory Networks of Plutella xylostella in Response to Metarhizium anisopliae Infection
Source: Insects. 2022 Oct 9;13(10):916. doi: 10.3390/insects13100916 (PMC9604237; doi:10.3390/insects13100916)

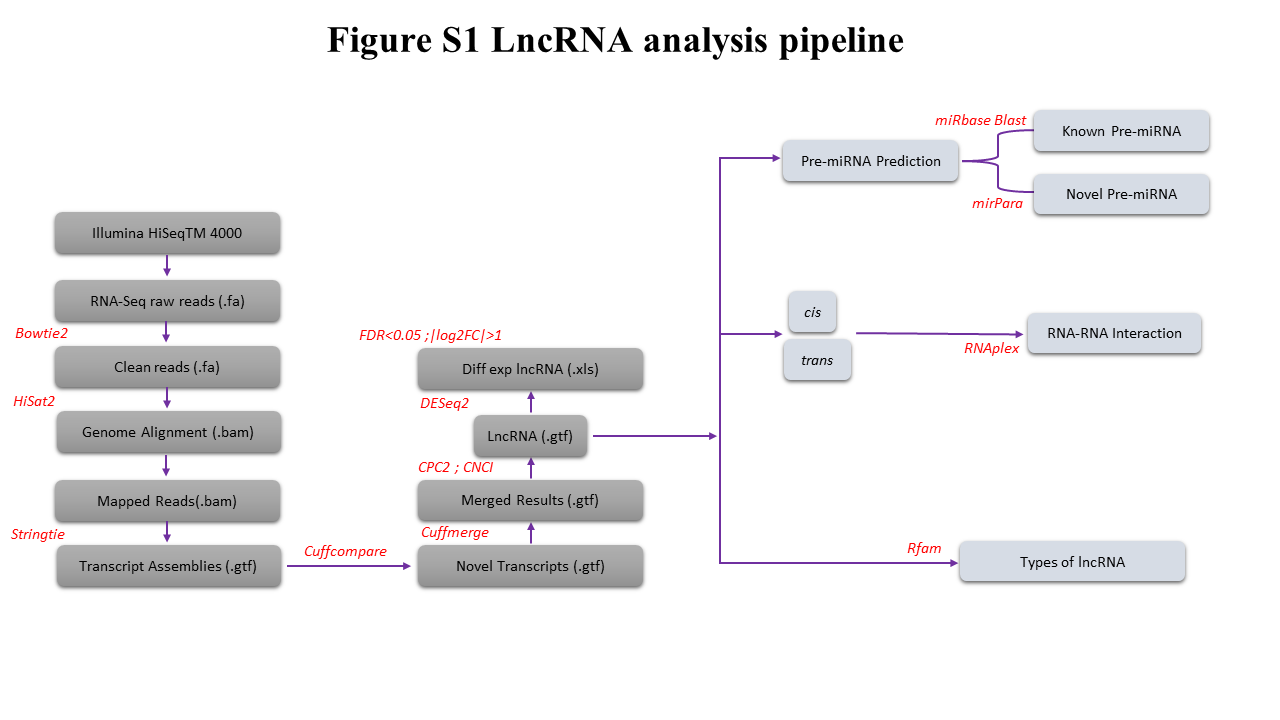

Supplement: Supplementary file 1 [file insects-13-00916-s001.zip › Figure S1 The lncRNA analysis pipeline.tif]
